# Association between polymorphisms in the adiponectin gene and cardiovascular disease: a meta-analysis

**DOI:** 10.1186/1471-2350-13-40

**Published:** 2012-05-28

**Authors:** Huan Zhang, Xingbo Mo, Yongchen Hao, Dongfeng Gu

**Affiliations:** 1 State Key Laboratory of Cardiovascular Disease, Department of Evidence Based Medicine and Division of Population Genetics, Fuwai Hospital, National Center of Cardiovascular Diseases, Chinese Academy of Medical Sciences and Peking Union Medical College, Beijing, 100037, China

**Keywords:** Adiponectin, Polymorphisms, Cardiovascular disease, Meta-analysis

## Abstract

**Background:**

Previous studies have examined the associations between polymorphisms of adiponectin gene (ADIPOQ) and cardiovascular disease (CVD), but those studies have been inconclusive. The aim of this study was to access the relationship between three single nucleotide polymorphisms (SNPs), +45 T > G (rs2241766), +276 G > T (rs1501299) and -11377 C > G (rs266729) in ADIPOQ and CVD.

**Methods:**

A comprehensive search was conducted to identify all studies on the association of ADIPOQ gene polymorphisms with CVD risk. The fixed and random effect pooled measures (i.e. odds ratio (OR) and 95% confidence interval (CI)) were calculated in the meta-analysis. Heterogeneity among studies was evaluated using Q test and the I^2^. Publication bias was estimated using modified Egger’s linear regression test.

**Results:**

Thirty-seven studies concerning the associations between the three polymorphisms of ADIPOQ gene and CVD risk were enrolled in this meta-analysis, including 6,398 cases and 10,829 controls for rs2241766, 8,392 cases and 18,730 controls for rs1501299 and 7,835 cases and 14,023 controls for rs266729. The three SNPs were significantly associated with CVD, yielding pooled ORs of 1.22 (95%CI: 1.07, 1.39; *P* = 0.004), 0.90 (95%CI: 0.83, 0.97; *P* = 0.007) and 1.09(95%CI: 1.01, 1.17; *P* = 0.032) for rs2241766, rs1501299 and rs266729, respectively. Rs2241766 and rs1501299 were significantly associated with coronary heart disease (CHD), yielding pooled ORs of 1.29 (95%CI: 1.09, 1.52; *P* = 0.004) and 0.89 (95%CI: 0.81, 0.99; *P* = 0.025), respectively. The pooled OR for rs266729 and CHD was 1.09 (95%CI: 0.99, 1.19; *P* = 0.090). Significant between-study heterogeneity was found in our meta-analysis. Evidence of publication bias was observed in the meta-analysis.

**Conclusions:**

The present meta-analysis showed that the associations between rs2241766, rs1501299 and rs266729 in the ADIPOQ and CVD were significant but weak. High quality studies are still needed to confirm the associations, especially for rs2241766.

## Background

Adiponectin is a 30-kDa protein that consists of an N-terminal collagenous domain and a C-terminal globular domain[1], with circulating levels ranging from 0.5 to 30 μg/ml and accounting for 0.05% of total plasma protein[2,3].

Adiponectin plays a role in preventing atherosclerosis. Epidemiology studies have found that adiponectin levels were associated with risk of cardiovascular disease (CVD). Some cross-sectional studies have demonstrated that hypoadiponectinemia was associated with the prevalence of CVD[4-7]. Prospective studies also found significant inverse association between adiponectin and CVD. Health Professionals Follow-up Study found that high plasma adiponectin levels were associated with lower risk of myocardial infarction (MI) over a follow-up period of 6 years among men without previous cardiovascular disease[8]. The Nurses’ Health Study recently found that high levels of total adiponectin were associated with lower risk of CVD among women during 14 years of follow-up [9].

There have been a number of studies reported that certain polymorphisms in the adiponectin coding gene ADIPOQ were strongly associated with adiponectin levels [10-13]. A recent genome wide association study (GWAS) identified that a single nucleotide polymorphism (SNP) rs266717 at the ADIPOQ locus demonstrated the strongest associations with adiponectin levels (*P*-combined = 9.2 × 10^−19^, n = 14,733) [14]. In that study, the authors also re-evaluated other SNPs in ADIPOQ and found that rs266729 and rs182052 showed significant association. Previous studies have examined the association of several polymorphisms in the ADIPOQ gene, including rs822395 (−4034A > C), rs822396 (−3964A > G), +2019delA, rs17300539 (−11391 G > A), rs266729 (−11377 C > G), rs2241766 (+45 T > G) and rs1501299 (+276 G > T), with CVD and subclinical CVD [15]. The three SNPs, rs266729, rs2241766 and rs1501299, were most widely studied.

Lacquemant et al. reported that rs2241766 was associated with an increased risk of coronary artery disease among patients with type 2 diabetes[16]. Bacci et al. reported that polymorphism rs1501299 was associated with a decreased coronary artery disease risk [17]. However, these early studies have been limited by the small sample size and case–control design. Subsequent researches on this issue reported different results. A large genetic association case–control study conducted by Chiodini et al. confirmed the association for rs1501299, but SNP rs2241766 showed no significant association [18]. The studies conducted by Pischon et al. also failed to confirm these associations [9]. The result for rs266729 has also been inconsistent [19-21].

Meta-analysis is a powerful tool for summarizing results from different studies by producing a single estimate of the major effect with enhanced precision. In this study, we conducted a meta-analysis to examine the associations between the three SNPs in the ADIPOQ gene and CVD.

## Methods

### Retrieval of published studies

Two independent reviewers (Zhang and Mo) conducted a systematic computerized literature search for papers published before February 12, 2012. PubMed, Embase and Wanfang databases were searched, using various combinations of keywords, such as ‘cardiovascular disease’ or ‘coronary heart disease’ or ‘coronary artery disease’ or ‘myocardial infarction’ or ‘stroke’ combined with ‘ADIPOQ’ or ‘APM1’ or ‘ACDC’ or ‘adiponectin’ and ‘polymorphism’ and ‘genetic association’, without language restriction. The full texts of the retrieved articles were read to decide whether information on the topic of interest should be included. The reference lists of the retrieved articles as well as those of review articles and previous meta-analyses on this topic were searched to identify other studies that were not identified initially. Articles were included in the meta-analysis if they examined the hypothesis that ADIPOQ polymorphisms were associated with CVD using case–control or cohort design, and had sufficient published data on the genotypes or allele frequencies for determining an estimate of relative risk (i.e. odds ratio (OR)) and confidence interval (CI). Our meta-analysis was conducted according to the Meta-analysis of Observational Studies in Epidemiology (MOOSE) guidelines [22].

### Data extraction

Two reviewers (Zhang and Mo) independently examined the retrieved articles using a data collection form, in order to extract the information needed. From each study, the following data were abstracted: first author, year of publication, country of the population studied, study subjects and main outcomes, the mean age and body mass index (BMI) and the percentage of men in case and control groups, polymorphisms tested, genotyping methods, the number of persons with different genotypes in cases and controls, and main results. Following data extraction, the reviewers checked for any discordance until a consensus was reached.

### Quality score assessment

Quality of studies was also independently assessed by the same two reviewers, using guidelines proposed by the NCI-NHGRI Working Group on Replication in Association Studies [23]. These guidelines provided a checklist of 53 conditions for authors, journal editors and referees to allow clear and unambiguous interpretation of the data and results of genome-wide and other genotype–phenotype association studies. The first 34 conditions were considered for quality assessment of each study in our meta-analysis. One score is assigned to each condition. If one study met a requirement, it gained 1 score and otherwise, it gained 0. The sum of the score for each study was described as total quality score.

### Statistical analysis

The OR was used to compare alleles between cases and controls. We computed the genetic contrast of the mutant alleles (G for rs2241766, T for rs1501299 and G for rs266729) versus the wildtype alleles. In secondary analyses, we calculated specific ORs according to the racial descent of subjects (separated analyses for Caucasian, East Asian, West Asian and African populations), study subjects (normal subjects or subjects with type 2 diabetes or other diseases), sample size (<1000 or ≥1000), genotyping methods (PCR-RFLP or Taqman genotyping assay or other). The association for coronary heart disease (CHD) was also examined. We assessed the presence of between-study heterogeneity using the chi-square based Cochran’s Q statistic. The inconsistency index I^2^ (ranging from 0 to 100%) was also calculated, where higher values of the index (I^2^ > 50%) indicate the existence of heterogeneity[24]. Publication bias was assessed with Egger regression test [25]. The pooled OR was calculated by the inverse-variance weighted method, and the significance of the pooled OR was tested by Z statistic. The combined ORs along with their 95% CIs were estimated using the fixed effects and random effects method. The random-effects method[26], which in the presence of heterogeneity, is more appropriate as it is prudent to take into account an estimate of the between-study variance (I^2^). On the other hand, the random effects hypothesis is appropriate for clinical trials but results in relatively reduced power for genetic association/GWAS detection of SNPs which show association in at least one study [27]. The random effects model has less power to detect effects than fixed effects in almost all situations [28]. To examine specific subsets in these studies, separate analyses were undertaken. This was achieved by performing a sensitivity analysis, in which an individual study was removed each time to assess the influence of each study. Likewise, a cumulative analysis was performed according to the ascending date of publication to identify the influence of the first published study on the subsequent publications and the evolution of the combined estimate over time [29]. For all analyses performed here, the statistical package Stata 10 (Stata Corporation, College Station, Texas, USA) was used. In all analyses statistically significant results were declared as those with a *P* value < 0.05, except for tests of publication bias where 0.1 was used as significant level.

## Results

**Figure 1:**
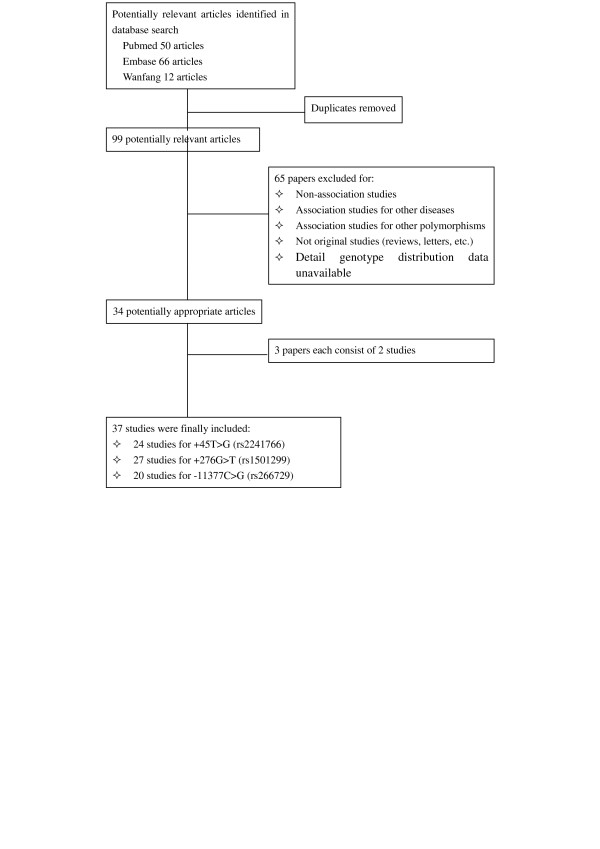
Flow diagram for study selection process in the meta-analysis of ADIPOQ gene polymorphisms and CVD.

After the literature searching and the subsequent screening, we came up with 34 research papers consisting of 37 case–control or cohort studies concerning the association of rs2241766 or rs1501299 or rs266729 polymorphisms with CVD (Figure 1). The detailed characteristics of each study were summarized, including authors, publication year, mean age, percentage of men, sample size, genotyping method and study population (Table 1). We also summarized the mean BMI in case and control groups and genotype data in case and control groups (Additional file 1: Table S1) and the main results of each study (Additional file 2: Table S2). Details of the quality score assessment were presented in Additional file 3: Table S3.

**Table 1:** Characteristics of the eligible studies included in the meta-analysis

**Study**	**Country**	**Year**	**Sample size**		**Mean age(Yr)**		**Percentage of men (%)**		**Study population**	**Outcome**	**Genotyping method**	**Quality score**
			**Case**	**Control**	**Case**	**Control**	**Case**	**Control**				
Lacquemant[16]	Switzerland	2003	107	181	/	/	/	/	T2D	CAD	Other	9
Lacquemant[16]	France	2003	55	134	/	/	/	/	T2D	CAD	Other	9
Bacci[17]	Italy	2004	142	234	64.0	60.0	64.1	43.2	T2D	CHD	Other	8
Ohashi [30]	Japan	2004	383	368	63.0	62.3	70.5	65.2	General	CAD	TaqMan	7
Stenvinkel [31]	America	2004	63	141	/	/	/	/	Renal disease	CVD	Other	7
Filippi [32]	Italy	2005	580	466	60.3	50.9	76.9	49.6	General	CAD	Other	9
Ru [33]	China	2005	131	136	51.3	50.6	56.2	52.9	General	CHD	TaqMan	6
Qi [21]	America	2005	239	640	59.6	55.0	100	100	T2D	CVD	TaqMan	10
Qi [34]	America	2006	285	704	47.0	44.0	0	0	T2D	CVD	TaqMan	10
Gable [35]	UK	2006	266	2727	56.0	56.6	100	100	General	CVD	PCR-RFLP	12
Gable [35]	UK	2006	530	564	56.0	56.6	100	100	General	MI	PCR-RFLP	12
Wang [36]	China	2006	120	131	/	/	/	/	General	CHD	PCR-RFLP	7
Hegener[37]	America	2006	341	341	60.2	60.1	100	100	General	MI	TaqMan	11
Hegener[37]	America	2006	259	259	62.1	61.7	100	100	General	Stroke	TaqMan	11
Jung[38]	Korea	2006	88	86	60.4	53.4	71.6	50	General	CAD	TaqMan	8
Pischon[39]	America	2007	1036	2071	62.9	62.8	51.7	51.7	General	CHD	TaqMan	11
Lu[40]	China	2007	131	135	58.4	60.7	68.9	62.6	General	CHD	PCR-RFLP	7
Liang[41]	China	2008	100	100	45.7	60.8	66.0	65.0	General	CHD	PCR-RFLP	6
Yamada[42]	Japan	2008	313	971	67.0	68.2	61.7	48.7	MetS	ACI	Other	9
Oguri[19]	Japan	2009	773	1114	64.8	68.3	77.2	50.8	MetS	MI	Other	10
Chang[43]	China	2009	600	718	63.8	51.1	78.3	53.4	General	CAD	PCR-RFLP	9
Foucan[44]	France	2009	57	159	68.0	63.0	51.0	36.0	T2D	CAD	TaqMan	7
Zhang[45]	China	2009	205	130	65.0	63.0	63.4	50.4	General	CHD	PCR-RFLP	8
Zhong[46]	China	2010	198	237	60.6	54.5	54.0	46.0	General	CAD	TaqMan	10
De Caterina[47]	Italy	2010	1864	1864	39.5	39.6	88.8	88.8	General	MI	Other	13
Xu[48]	China	2010	153	73	66.3	66.3	53.6	53.4	General	CHD	PCR-RFLP	8
Al-Daghri[49]	Saudi Arabia	2010	123	295	69.4	60.7	60	70	T2D	CAD	PCR-RFLP	8
Prior[20]	UK	2010	85	298	71.0	68.2	63.6	50.6	General	CHD	PCR-RFLP	7
Chiodini[18]	Italy	2010	503	503	56.5	54.7	89.3	95.8	General	MI	TaqMan	10
Rodriguez[50]	Spain	2010	119	555	/	/	/	/	RA	CVD	TaqMan	9
Leu[51]	China	2010	80	3330	59.1	50.0	52.5	45.3	General	stroke	Other	10
Liu[52]	China	2010	302	338	65.7	64.4	63.9	62.1	General	stroke	PCR-RFLP	9
Chen[53]	China	2010	357	345	63.6	53.7	60.2	60.9	General	stroke	TaqMan	8
Sabouri[54]	UK	2011	329	106	58.4	47.6	64.1	56.3	General	CAD	PCR-RFLP	8
Esteghamati[55]	Iran	2011	144	127	61.1	51.1	38.6	55.9	General	CAD	PCR-RFLP	10
Boumaiza[56]	Tunisia	2011	212	104	60.6	59.4	69.3	55.8	General	CAD	PCR-RFLP	10
Katakami[57]	Japan	2012	213	2424	58.1	54.6	66.2	60.7	T2D	CVD	Other	11

ACI = atherothrombotic cerebral infarction; CAD = coronary artery disease; CHD = coronary heart disease; CVD = cardiovascular disease; MI = myocardial infarction; MetS = metabolic syndrome; RA = rheumatoid arthritis; T2D = type 2 diabetes.

### Meta-analysis for rs2241766 polymorphism

The 24 retrieved studies that investigated the association of rs2241766 with CVD contained information about 6,398 cases and 10,829 control subjects (Table 2). The pooled frequency of the minor G allele in controls was 15.7%.

**Table 2:** Meta-analysis of ADIPOQ gene polymorphisms and CVD

	**+45 T > G**					**+276 G > T**					**−11377 C > G**				
	**No. studies**	**Sample size**	**Per allele risk**		**I^2^%**	**No. studies**	**Sample size**	**Per allele risk**		**I^2^%**	**No. studies**	**Sample size**	**Per allele risk**		**I^2^%**
		**Case/control**	**OR(95%CI)**	**P**			**Case/control**	**OR(95%CI)**	**P**			**Case/control**	**OR(95%CI)**	**P**	
**Overall**	24	6398/10829	1.22(1.07-1.39)	0.004	74.2	27	8392/18730	0.90(0.83-0.97)	0.007	58.0	20	7835/14023	1.09(1.01-1.17)	0.032	53.6
**CHD**	17	4685/5881	1.29(1.09-1.52)	0.004	76.9	18	6585/7760	0.89(0.81-0.99)	0.025	65.1	11	5687/7431	1.09(0.99-1.19)	0.090	51.8
**Population**															
European origin	12	3751/8269	1.10(0.94-1.27)	0.226	63.1	15	6306/11254	0.95(0.89-1.02)	0.155	33.8	14	5729/10924	1.01(0.94-1.08)	0.880	26.1
East Asian	7	1813/1766	1.19(0.91-1.56)	0.209	82.6	9	1637/6948	0.83(0.68-1.02)	0.074	73.3	6	2106/3099	1.29(1.18-1.42)	<0.001	0
West Asian	3	565/531	2.07(1.33-3.22)	0.001	59.5	2	237/424	0.75(0.41-1.36)	0.337	81.4	0				
African	2	269/263	1.38(0.79-2.41)	0.257	36.7	1	212/104	0.75(0.53-1.07)	0.110	/	0				
**Study subjects**															
Normal subjects	17	5501/8723	1.20(1.03-1.41)	0.018	78.7	17	6949/13367	0.92(0.84-1.01)	0.082	63.3	12	5897/9627	1.09(1.00-1.19)	0.042	46.6
Subjects with T2D	6	834/1965	1.18(0.90-1.54)	0.222	52.2	8	1261/4667	0.88(0.76-1.02)	0.098	47.0	4	672/1615	0.87(0.75-1.01)	0.07	0
Subjects with MetS	0					0					2	1084/2085	1.29(1.15-1.46)	<0.001	0
Subjects with RD	1	63/141	2.06(1.10-3.83)	0.023	/	1	63/141	0.64(0.39-1.03)	0.063	/	1	63/141	1.45(0.90-2.32)	0.129	/
Subjects with RA	0					1	119/555	0.79(0.57-1.09)	0.151	/	1	119/555	1.01(0.73-1.40)	0.941	/
**Sample size**															
More than 1000	5	2911/6409	1.01(0.80-1.28)	0.912	84.5	8	5006/13809	0.93(0.84-1.04)	0.216	65.2	7	5274/9800	1.13(1.03-1.25)	0.014	62.5
Less than 1000	19	3487/4420	1.30(1.12-1.52)	0.001	63.9	19	3386/4921	0.87(0.78-0.97)	0.015	56.0	13	2561/4223	1.04(0.93-1.18)	0.488	49.6
**Genotyping method**															
TaqMan	9	3101/4986	1.13(0.94-1.37)	0.188	70.8	10	3362/5571	0.93(0.85-1.01)	0.098	31.6	9	3284/5572	1.00(0.91-1.10)	0.959	33.8
PCR-RFLP	11	2942/5167	1.25(1.01-1.56)	0.043	80.6	9	1958/4516	0.80(0.68-0.94)	0.006	63.1	5	1388/4057	1.19(1.03-1.39)	0.023	43.2
Other	4	355/676	1.44(0.99-2.11)	0.058	54.3	8	3072/8643	0.97(0.83-1.14)	0.728	64.7	6	3163/4394	1.15(0.98-1.35)	0.090	64.0

MAF = minor allele frequency; OR = odds ratio; CI = confidence interval; NA = not available; T2D = type 2 diabetes; MetS = metabolic syndrome; RD = renal disease; RA = rheumatoid arthritis; I^2^: The inconsistency index for between-studies heterogeneity, where higher values of the index (I2 > 50%) indicate the existence of heterogeneity.

**Figure 2:**
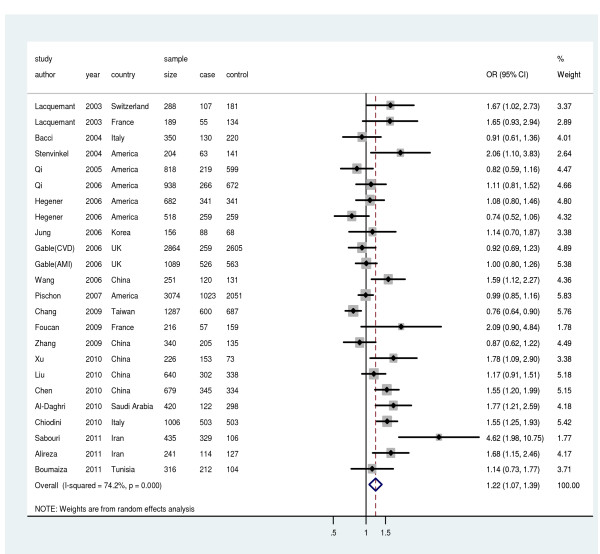
** Meta-analysis for the relationship between rs2241766 and CVD risk.** Year represents publish year. The solid squares represent odds ratios (ORs) from individual studies; the diamonds are shown as overall effect. The combined ORs along with their 95% CIs were in the contrast of G allele vs. T allele and estimated using the random-effects method.

Figure 2 showed that the combined ORs (fixed-effects and random-effects method) for the rs2241766G allele on CVD were 1.12 (95%CI: 1.05, 1.19; *P* < 0.0001) and 1.22 (95%CI: 1.07, 1.39; *P* = 0.004). There was a significant between-study heterogeneity as indicated by the *P* value of the corresponding test (*P* < 0.001) and the value of the I^2^ index (I^2^ =74.2%). The sensitivity analysis revealed that there was not a single study influencing the result significantly. Cumulative analysis found the influence of the first published study on the subsequent publications and the evolution of the combined estimate over time. Lacquemant et al. reported the significant association in 2003 [14], while the subsequent publications added to the meta-analysis, the significance disappeared. The overall estimation became significant after the large study reported by Chiodini et al. in 2010 included in the analysis [16]. Evidence of publication bias was found in the studies (Egger’s test, *P* = 0.007). The association for CHD alone was also significant, with an OR of 1.29(95%CI: 1.09, 1.52) (Table 2).

### Meta-analysis for rs1501299 polymorphism

Twenty-seven studies investigating the association of rs1501299 with CVD were enrolled in this meta-analysis, containing about 8,392 cases and 18,730 control subjects (Table 2). The pooled frequency of the T allele in control groups was 28.3%.

**Figure 3:**
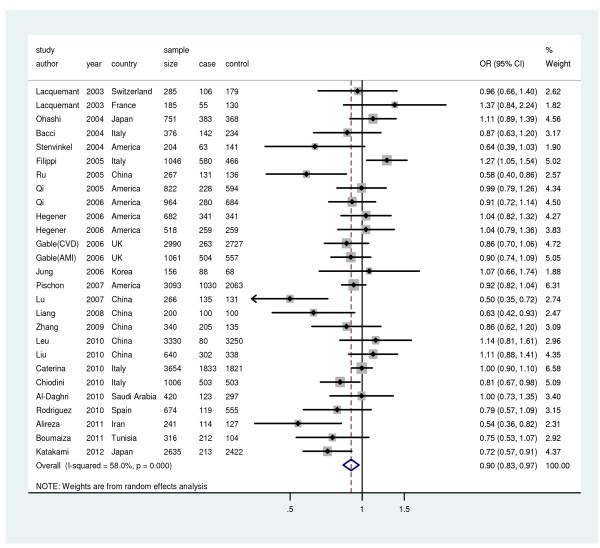
** Meta-analysis for the relationship between rs1501299 and CVD risk.** Year represents publish year. The solid squares represent odds ratios (ORs) from individual studies; the diamonds are shown as overall effect. The combined ORs along with their 95% CIs were in the contrast of T allele vs. G allele and estimated using the fixed-effects method.

A significant association was observed between the rs1501299T allele and risk of CVD (Figure 3), yielding overall ORs (fixed-effects and random-effects method) of 0.93 (95%CI: 0.89, 0.97; *P* = 0.001) and 0.90 (95% CI: 0.83, 0.97; *P* = 0.007). Significant heterogeneity was observed (I^2^ = 58.0%, *P* < 0.001). The sensitivity analysis revealed that there was not a single study influencing the result significantly. Cumulative analysis did not find the influence of the first published study on the subsequent publications and the evolution of the combined estimate over time. There was publication bias in the studies (Egger’s test, *P* = 0.077). The combined OR for rs1501299 and CHD was 0.89(95%CI: 0.81, 0.99) (Table 2).

### Meta-analysis for rs266729 polymorphism

Twenty studies including about 7,835 cases and 14,023 controls were enrolled in this meta-analysis for the association between rs266729 and CVD (Table 2). For all the studies included, we found that the control groups were in HWE. The pooled frequency of the G allele was 24.8% in the control groups.

**Figure 4:**
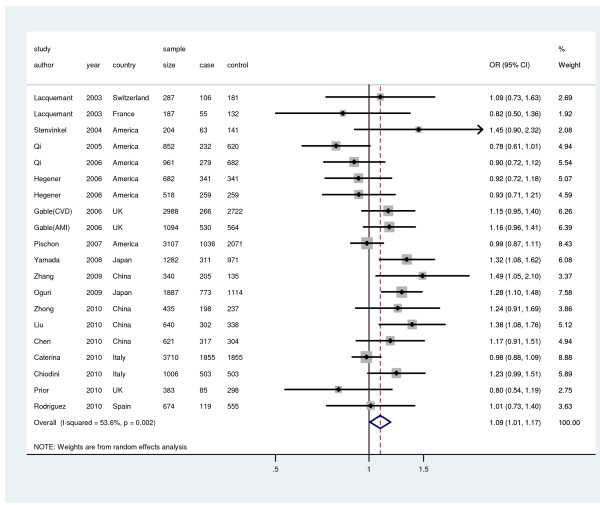
** Meta-analysis for the relationship between rs266729 and CVD risk**. Year represents publish year. The solid squares represent odds ratios (ORs) from individual studies; the diamonds are shown as overall effect. The combined ORs along with their 95% CIs were in the contrast of G allele vs. C allele and estimated using the random-effects method.

The association for rs266729 and CVD was significant, with ORs (fixed-effects and random-effects method) of 1.07 (95%CI: 1.02, 1.13; *P* = 0.003) and 1.09(95%CI: 1.01, 1.17; *P* = 0.032), and significant heterogeneity (I^2^ = 53.6%, *P* = 0.002) (Figure 4). By performing subgroup analyses we found that the East Asian studies indicated significant association (OR = 1.29, 95% CI: 1.18, 1.42). Heterogeneity disappeared in this subgroup analysis, I^2^ indexes equal to 26.1% and 0 for European subgroup and Asian subgroup, respectively (Table 2). The sensitivity analysis revealed that there was not a single study influencing the result significantly. Cumulative analysis did not find the influence of the first published study on the subsequent publications and the evolution of the combined estimate over time. The results of Egger’s test did not suggest publication bias in the studies, *P* = 0.624. No significant association was found between rs266729 and the risk of CHD (OR = 1.09, 95% CI: 0.99, 1.19, random-effects method), with significant between-study heterogeneity (I^2^ = 51.8%, *P* = 0.023) (Table 2).

## Discussion

The present meta-analysis, involving 6 to 8 thousand cases and more than 10 thousand controls for each polymorphism, provides a clear indication of significant associations between the three SNPs, rs2241766, rs1501299 and rs266729, in ADIPOQ and CVD. The findings of this study suggest that the rs2241766G allele and rs266729G allele increase odds of CVD, while the rs1501299T allele decreases. Rs2241766 and rs1501299 are significantly associated with CHD. However, the results were nearly “crude” ones, other important risk factors for CVD or CHD might diminish the significance of the results, so the associations were significant but weak. Nevertheless, the magnitude of these associations is in the range of all positive associations found with SNPs in multifactorial polygenic disorders, even with the “top ten” SNPs from GWAS.

Qi et al. have conducted a meta-analysis to summarize the association between rs1501299 and CVD risk among diabetic patients [34]. 827 CVD cases and 1,887 CVD-free control subjects were included in their meta-analysis. They found that the minor allele T homozygote was significantly associated with ~45% reduction in CVD risk. A recent meta-analysis for rs1501299 and CVD also reported the protect effect of rs1501299T allele in type 2 diabetes population [58]. Our meta-analysis included more studies and collected more information than previous meta-analyses. We also evaluated the association for the SNP rs1501299 under a recessive model among type 2 diabetic patients. The result was very similar to that of previous meta-analyses, with an OR of 0.71(95%CI: 0.56, 0.91). Previous meta-analysis did not evaluate the associations between rs2241766 and rs266729 and CVD risk. According to our results, significant associations were also observed for these two important SNPs.

The findings of our study may shed light on the underlying disease mechanism in CVD. Previous studies and meta-analysis demonstrated that rs1501299T carriers had higher adiponectin levels [10,59], which were associated with lower odds of CVD [8,9,60,61]. Besides, these SNPs were reported to be associated with many conventional CVD risk factors, such as hypertension, obesity, diabetes mellitus, insulin restriction and metabolic syndrome. The HOMA-IR (homeostasis model assessment of insulin resistance) index was higher in rs1501299GG carriers as compared with TG and TT subjects, indicating higher insulin sensitivity in carriers of allele T, the same allele that showed an association with increased adiponectin levels [59]. Rs1501299 was also found to be associated with the presence of hypertension in metabolic syndrome individuals [62]. The rs266729G allele was reported to be significantly associated with higher odds of hypertension in Hong Kong Chinese, after adjusting for covariates. In stepwise multiple logistic regression, this SNP was a significant independent risk factor of hypertension, together with age, body mass index, triglycerides, and insulin resistance index. Also, rs266729 was significantly associated with adiponectin level after adjusting for covariates [63]. Previous meta-analysis also detected a significant association between rs266729 and an increased risk of T2D [64]. As mentioned above, the effect of rs1501299 and rs266729 on CVD risk might be regulated by adiponectin concentrations, as well as other CVD risk factors. Nevertheless, these particular SNPs are far from being the most influent on adiponectin levels and CVD risk factors, and the association is weak and/or controversial, especially for rs266729 and rs2241766.

Several potential limitations of our study should be noted. First, significant between-study heterogeneities were observed in our study, we tried to explored the source of heterogeneities with factors such as ethnicity, study subjects, sample size, genotyping methods in the subgroup analyses. However, for rs2241766 and rs1501299 polymorphisms, significant heterogeneities still remained, only the heterogeneity for rs266729 disappeared by performing subgroup analyses according to ethnicity. Second, as significant publication bias was observed in the analysis of rs2241766, the association for this SNP still needs to be confirmed in future studies. Third, the eligible studies in our research were mainly from Asia and Europe, data of other populations, like African, was limited. Fourth, because we did not have access to individual data, we could not control for population stratification, nor could we adjust for variables in possible intermediate pathways. Finally, we should realize that the results might be distorted by potential weakness and biases of genetic association studies, such as genotyping error, phenotype misclassification, population stratification, gene-gene or gene-environment interactive effect, and selective reporting biases [29,65].

Further studies should be conducted to confirm the functional variants. More studies are needed to elucidate the complete range of the signal transduction pathways that the variant is implicated in, and thus, throw light in the underlying molecular mechanisms that confer susceptibility to CVD. The particular polymorphism associated with CVD itself may not play a functional role, but rather it may be located physically close to the actual disease-predisposing gene.

In addition to the genetic markers described in this meta-analysis, many other polymorphisms have been studied. Those polymorphisms may be candidates for a multivariate analysis. Future haplotypic approaches and further haplotype-based meta-analyses will provide more powerful and informative results than current single genotype-based data.

## Conclusions

The present meta-analysis showed that the associations between rs2241766, rs1501299 and rs266729 in the ADIPOQ and CVD were significant but weak. The rs2241766G allele and rs266729G allele increase risk of CVD, while the rs1501299T allele decreases. High quality studies are still needed to confirm the associations, especially for rs2241766.

## Abbreviations

ACI: atherothrombotic cerebral infarction; ADIPOQ: adiponectin gene; CAD: coronary artery disease; CHD: coronary heart disease; CVD: cardiovascular disease; CI: confidence interval; HWE: hardy-Weinberg equilibrium; MAF: Minor Allele Frequency; MetS: Metabolic syndrome; MI: myocardial infarction; NA: Not available; OR: odds ratio; RA: Rheumatoid arthritis; RD: Renal disease; T2D: Type 2 diabetes.

## Competing interests

The authors declare that they have no conflict of interest.

## Authors’ contributions

HZ and DFG conceived of the study, and participated in its design and coordination. HZ, XBM and YCH carried out the literature searching and data extraction, independently. XBM and HZ performed the statistic analysis and draft the manuscript. All authors read and approved the final manuscript.

## Pre-publication history

The pre-publication history for this paper can be accessed here:

http://www.biomedcentral.com/1471-2350/13/40/prepub

## Supplementary Material

Additional file 1 Table S1. Characteristics of the eligible studies included in the meta-analysis. Click here for file

Additional file 2 Table S2. Main results of each study included in the meta-analysis. Click here for file

Additional file 3 Table S3. Quality assessment for each study included in this meta-analysis. Click here for file
