# Supplementary material for: Association between polymorphisms in the adiponectin gene and cardiovascular disease: a meta-analysis
Source: BMC Med Genet. 2012 May 28;13:40. doi: 10.1186/1471-2350-13-40 (PMC3413575; doi:10.1186/1471-2350-13-40)
Supplement: Additional file 1 — Table S1. Characteristics of the eligible studies included in the meta-analysis. [file 1471-2350-13-40-S1.doc]

Supplement table 1 -Characteristics of the eligible studies included in the meta-analysis

| Study | Country | Year | Mean age(Yr) | | Percentage of men (%) | | BMI | | | rs2241766  (case/control) | | | rs1501299  (case/control) | | | | rs266729  (case/control) | | | |
| --- | --- | --- | --- | --- | --- | --- | --- | --- | --- | --- | --- | --- | --- | --- | --- | --- | --- | --- | --- | --- |
|  |  |  | Case | Control | Case | Control | Case | Control | | TT | TG | GG | GG | GT | TT | CC | | CG | GG |  |
| Lacquemant | Switzerland | 2003 | / | / | / | / | / | | / | 76/145 | 27/34 | 4/2 | 57/96 | 40/65 | 9/18 | 57/109 | | 48/64 | 1/8 |  |
| Lacquemant | France | 2003 | / | / | / | / | / | | / | 33/104 | 21/23 | 1/7 | 25/73 | 26/50 | 4/7 | 32/65 | | 17/54 | 6/13 |  |
| Bacci | Italy | 2004 | 64.0 | 60.0 | 64.1 | 43.2 | 29.0 | | 30.0 | 90/149 | 35/60 | 5/11 | 70/118 | 65/88 | 7/28 |  | |  |  |  |
| Ohashi | Japan | 2004 | 63.0 | 62.3 | 70.5 | 65.2 | 24.1 | | 23.8 |  |  |  | 185/190 | 164/149 | 34/29 |  | |  |  |  |
| Stenvinkel | America | 2004 | / | / | / | / | / | | / | 44/119 | 17/19 | 2/3 | 38/62 | 20/65 | 5/14 | 29/88 | | 31/43 | 3/10 |  |
| Filippi | Italy | 2005 | 60.3 | 50.9 | 76.9 | 49.6 | 27.0 | | 28.6 |  |  |  | 287/266 | 241/167 | 52/33 |  | |  |  |  |
| Ru | China | 2005 | 51.3 | 50.6 | 56.2 | 52.9 | / | | / |  |  |  | 79/64 | 46/55 | 6/17 |  | |  |  |  |
| Qi | America | 2005 | 59.6 | 55.0 | 100 | 100 | 27.8 | | 27.6 | 170/440 | 49/159 | 0/0 | 105/293 | 111/249 | 12/52 | 138/333 | | 85/243 | 9/44 |  |
| Qi | America | 2006 | 47.0 | 44.0 | 0 | 0 | 29.1 | | 27.7 | 204/529 | 62/143 | 0/0 | 159/374 | 104/258 | 17/52 | 158/355 | | 102/283 | 19/44 |  |
| Gable | UK | 2006 | 56.0 | 56.6 | 100 | 100 | 26.7 | | 26.2 | 204/2022 | 53/548 | 2/35 | 155/1511 | 96/1038 | 12/178 | 129/1480 | | 120/1063 | 17/179 |  |
| Gable | UK | 2006 | 56.0 | 56.6 | 100 | 100 | 26.7 | | 26.2 | 360/384 | 154/168 | 12/11 | 266/289 | 216/225 | 22/43 | 278/329 | | 217/197 | 35/38 |  |
| Wang | China | 2006 | / | / | / | / | / | | / | 28/49 | 65/64 | 27/18 |  |  |  |  | |  |  |  |
| Hegener | America | 2006 | 60.2 | 60.1 | 100 | 100 | 25.5 | | 24.9 | 241/252 | 95/80 | 5/9 | 183/181 | 134/143 | 24/17 | 198/188 | | 123/133 | 20/20 |  |
| Hegener | America | 2006 | 62.1 | 61.7 | 100 | 100 | 25.3 | | 24.8 | 203/188 | 52/64 | 4/7 | 137/136 | 98/104 | 24/19 | 123/134 | | 128/98 | 8/27 |  |
| Jung | Korea | 2006 | 60.4 | 53.4 | 71.6 | 50 | 25.4 | | 25.2 | 41/34 | 40/30 | 7/4 | 38/31 | 43/32 | 7/5 |  | |  |  |  |
| Pischon | America | 2007 | 62.9 | 62.8 | 51.7 | 51.7 | 26.5 | | 25.6 | 775/1560 | 231/447 | 17/44 | 555/1039 | 393/866 | 82/158 | 574/1166 | | 408/767 | 54/138 |  |
| Lu | China | 2007 | 58.4 | 60.7 | 68.9 | 62.6 | 25.6 | | 25.6 |  |  |  | 74/47 | 52/60 | 9/24 |  | |  |  |  |
| Liang | China | 2008 | 45.7 | 60.8 | 66.0 | 65.0 | 22.4 | | 21.5 |  |  |  | 29/16 | 64/67 | 7/17 |  | |  |  |  |
| Yamada | Japan | 2008 | 67.0 | 68.2 | 61.7 | 48.7 | 24.5 | | 25.3 |  |  |  |  |  |  | 163/575 | | 120/346 | 28/50 |  |
| Oguri | Japan | 2009 | 64.8 | 68.3 | 77.2 | 50.8 | 25.3 | | 25.4 |  |  |  |  |  |  | 397/675 | | 336/379 | 40/60 |  |
| Chang | China | 2009 | 63.8 | 51.1 | 78.3 | 53.4 | 25.0 | | 25.0 | 316/309 | 238/299 | 46/79 |  |  |  |  | |  |  |  |
| Zhang | China | 2009 | 65.0 | 63.0 | 63.4 | 50.4 | 24.9 | | 24.2 | 106/61 | 85/66 | 14/8 | 103/62 | 85/59 | 17/14 | 94/78 | | 87/47 | 24/10 |  |
| Zhong | China | 2009 | 60.6 | 54.5 | 54.0 | 46.0 | 25.0 | | 24.4 |  |  |  |  |  |  | 110/146 | | 72/76 | 16/15 |  |
| Foucan | France | 2009 | 68.0 | 63.0 | 51.0 | 36.0 | 29.0 | | 29.0 | 47/146 | 10/13 | 0/0 |  |  |  |  | |  |  |  |
| Caterina | Italy | 2010 | 39.5 | 39.6 | 88.8 | 88.8 | / | | / |  |  |  | 926/906 | 746/767 | 161/148 | 1076/1063 | | 671/684 | 108/108 |  |
| Xu | China | 2010 | 66.3 | 66.3 | 53.6 | 53.4 | 23.6 | | 23.2 | 78/50 | 75/23 | 0/0 |  |  |  |  | |  |  |  |
| Al-Daghri | Saudi Arabia | 2010 | 69.4 | 60.7 | 60 | 70 | 32.2 | | 30.3 | 77/220 | 35/72 | 10/6 | 47/111 | 57/142 | 19/44 |  | |  |  |  |
| Prior | UK | 2010 | 71.0 | 68.2 | 63.6 | 50.6 | 25.0 | | 25.0 |  |  |  |  |  |  | 46/158 | | 38/114 | 1/26 |  |
| Chiodini | Italy | 2010 | 56.5 | 54.7 | 89.3 | 95.8 | 26.4 | | 26.4 | 358/359 | 136/126 | 9/18 | 259/239 | 203/198 | 41/66 | 295/321 | | 177/160 | 31/22 |  |
| Rodriguez | Spain | 2010 | / | / | / | / | / | | / |  |  |  | 69/287 | 44/224 | 6/44 | 67/327 | | 46/188 | 6/40 |  |
| Leu | China | 2010 | 59.1 | 50.0 | 52.5 | 45.3 | 25.0 | | 24.3 |  |  |  | 39/1750 | 35/1261 | 6/239 |  | |  |  |  |
| Liu | China | 2010 | 65.7 | 64.4 | 63.9 | 62.1 | 24.5 | | 24.3 | 157/187 | 123/136 | 22/15 | 139/164 | 128/142 | 35/32 | 144/189 | | 125/128 | 33/21 |  |
| Chen | China | 2010 | 63.6 | 53.7 | 60.2 | 60.9 | / | | / | 192/221 | 117/95 | 36/18 |  |  |  | 174/176 | | 108/104 | 35/24 |  |
| Sabouri | UK | 2011 | 58.4 | 47.6 | 64.1 | 56.3 | 27.6 | | 27.5 | 253/100 | 74/6 | 2/0 |  |  |  |  | |  |  |  |
| Alireza | Iran | 2011 | 61.1 | 51.1 | 38.6 | 55.9 | 28.5 | | 29.6 | 48/68 | 41/46 | 25/13 | 76/63 | 30/47 | 8/17 |  | |  |  |  |
| Boumaiza | Tunisia | 2011 | 60.6 | 59.4 | 69.3 | 55.8 | 31.1 | | 33.3 | 145/75 | 57/24 | 10/5 | 105/45 | 84/41 | 23/18 |  | |  |  |  |
| Katakami | Japan | 2012 | 58.1 | 54.6 | 66.2 | 60.7 | / | | / |  |  |  | 129/1229 | 71/975 | 13/218 |  | |  |  |  |
