# Supplementary material for: Association between polymorphisms in the adiponectin gene and cardiovascular disease: a meta-analysis
Source: BMC Med Genet. 2012 May 28;13:40. doi: 10.1186/1471-2350-13-40 (PMC3413575; doi:10.1186/1471-2350-13-40)
Supplement: Additional file 2 — Table S2. Main results of each study included in the meta-analysis. [file 1471-2350-13-40-S2.doc]

**Supplement table 2 -Main results of each study included in the meta-analysis**

| **Study** | **Population** | **Polymorphism** | **Study subjects** | **Sample Size**  (case/control) | **Outcome** | **Genotyping Method** | **MAF** | **HWE** | **Genetic model** | **RESULT**  **P-value or OR (95%CI)** |
| --- | --- | --- | --- | --- | --- | --- | --- | --- | --- | --- |
| Lacquemant  2003 | European  Switzerland | rs2241766 | CAD patients and controls with T2D | 107/181 | CAD | Other :  PCR-direct sequencing  Or  the LightCycler technology | 0.10 | Yes | Genotypic | P=0.1 |
|  |  | rs1501299 |  | 106/179 |  |  | 0.28 | Yes | Genotypic | P=0.9 |
|  |  | rs266729 |  | 106/181 |  |  | 0.22 | Yes | Genotypic | P=0.09 |
| Lacquemant  2003 | European  France | rs2241766 | CAD patients and controls with T2D | 55/134 | CAD |  | 0.14 | Yes | Dominant | 2.3(1.2-4.5) |
|  |  | rs1501299 |  | 55/130 |  |  | 0.25 | Yes | Genotypic | P=0.4 |
|  |  | rs266729 |  | 55/132 |  |  | 0.30 | Yes | Genotypic | P=0.4 |
| Bacci  2004 | European | rs2241766 | CHD patients and controls with T2D | 142/234 | CHD | Other | 0.19 | NR | Genotypic | P=0.48 |
|  |  | rs1501299 |  |  |  |  | 0.31 | NR | Recessive | 0.30(0.12-0.76) |
| Stenvinkel  2004 | European | rs2241766 | CVD patients and controls with renal disease | 63/141 | CVD | Other  Pyrosequencing | 0.09 | Yes |  |  |
|  |  | rs1501299 |  |  |  |  | 0.33 | Yes |  |  |
|  |  | rs266729 |  |  |  |  | 0.22 | Yes | CC vs. GC | P<0.05 |
| Filippi  2005 | European | rs1501299 | CAD patients and normal controls | 580/466 | CAD | Other : Real-time PCR with LightCycler hybridization probes | 0.25 | Yes | Dominant | 20.7(1.05-4.07) |
| Qi  2005 | European | rs2241766 | CVD patients and controls with T2D | 239/640 | CVD | TaqMan genotyping assays  ABI 7900HT | 0.13 | Yes | Dominant | 0.77(0.51-1.16) |
|  |  | rs1501299 |  |  |  |  | 0.28 | Yes | Recessive | 0.38(0.18-0.79) |
|  |  | rs266729 |  |  |  |  | 0.27 | Yes | Additive  CG vs. CC  GG vs. CC | 0.90(0.63-1.29)  0.55(0.24-1.22) |
| Qi  2006 | European | rs2241766 | CVD patients and controls with T2D | 285/704 | CVD | TaqMan genotyping assays  ABI 7900HT | 0.11 | Yes | Dominant | 1.22(0.84-1.76) |
|  |  | rs1501299 |  |  |  |  | 0.33 | Yes | Additive  GT vs. GG  TT vs. GG | 0.94(0.68-1.29)  0.65(0.34-1.23) |
|  |  | rs266729 |  |  |  |  | 0.27 | Yes | Additive  CG vs. CC  GG vs. CC | 0.76(0.55-1.04)  0.93(0.50-1.72) |
| Gable  (NPSH II)  2006 | European  cohort | rs2241766 | CVD patients and normal controls | 259/2605 | CVD | PCR-RFLP | 0.12 | Yes | Genotypic | P=0.56 |
|  |  | rs1501299 |  | 263/2727 |  |  | 0.26 | Yes | Genotypic | P=0.26 |
|  |  | rs266729 |  | 266/2722 |  |  | 0.26 | Yes | Dominant | 1.33（1.05-1.70） |
| Gable  2006 | European | rs2241766 | MI patients and normal controls | 526/563 | MI | PCR-RFLP | 0.17 | Yes | Dominant | P=0.87 |
|  |  | rs1501299 |  | 504/557 |  |  | 0.28 | Yes | Dominant | P=0.96 |
|  |  | rs266729 |  | 530/564 |  |  | 0.24 | Yes | Dominant | 1.30(1.01-1.68) |
| Hegener  2006 | European | rs2241766 | MI, stroke patients and normal controls | 341/341  259/259 | MI  Stroke | TaqMan genotyping assays  ABI 7900HT | 0.14 | Yes | Genotypic  Allelic | P=0.26  P=0.59 |
|  |  | rs1501299 |  |  |  |  | 0.26 | Yes | Genotypic  Allelic | P=0.48  P=0.76 |
|  |  | rs266729 |  |  |  |  | 0.25 | Yes | Genotypic  Allelic | P=0.72  P=0.59 |
| Pischon  2007 | European | rs2241766 | CHD patients and normal controls | 1023/2051 | non-fatal MI or fatal CHD | TaqMan genotyping assays  ABI 7900HT | 0.13 | Yes |  |  |
|  |  | rs1501299 |  | 1030/2063 |  |  | 0.29 | Yes | Dominant | P=0.33 |
|  |  | rs266729 |  | 1036/2071 |  |  | 0.25 | Yes |  |  |
| Foucan  2009 | African Caribbean | rs2241766 | CAD patients and controls with T2D | 57/159 | CAD | TaqMan allelic discrimination | 0.06 | Yes | Dominant | 3.27(1.11-9.65) |
| Caterina  2010 | European | rs1501299 | MI patients and normal controls | 1864/1864 | early-onset MI | Other: Sequenom massarray | 0.29 | Yes | Additive  TG vs. GG  TT vs. GG | 1(0.90-1.10)  1(0.81-1.22) |
|  |  | rs266729 |  |  |  |  | 0.24 | Yes | Additive  CG vs. CC  GG vs. CC | 0.98(0.88-1.09)  0.96(0.71-1.28) |
| Persson  2010 | European | rs1501299 | MI patients and normal controls | 244/244 | MI | TaqMan genotyping assays  ABI 7000 | 0.30 | Yes | Recessive | 0.90(0.47-1.72) |
|  |  | rs266729 |  |  |  |  | 0.27 |  | Recessive | 1.36(0.63-2.97) |
| Prior  2010 | European | rs266729 | CHD patients and normal controls | 85/298 | CHD | PCR-RFLP | 0.27 | Yes | Dominant | 7.3(1.0-55.0) |
| Rodriguez  2010 | European | rs1501299 | CVD patients and controls with rheumatoid arthritis | 119/555 | CVD | TaqMan genotyping assays  ABI 7900HT | 0.28 | Yes | Allelic | 0.79(0.56-1.10) |
|  |  | rs266729 |  |  |  |  | 0.24 | Yes | Allelic | 1.01(0.72-1.42) |
| Chiodini  2010 | European | rs2241766 | MI patients and normal controls | 503/503 | MI | TaqMan genotyping assays  ABI 7900HT | 0.16 | Yes |  |  |
|  |  | rs1501299 |  |  |  |  | 0.33 | No | Recessive | 0.58 |
|  |  | rs266729 |  |  |  |  | 0.20 | Yes |  |  |
| Ohashi  2004 | East Asian | rs1501299 | CAD patients and normal controls | 383/368 | CAD | TaqMan genotyping assays  ABI Prism7200 | 0.28 | NR |  | Not significant |
| Ru  2005 | East Asian | rs1501299 | CHD patients and normal controls | 131/136 | CHD | TaqMan genotyping assays  ABI Prism377 | 0.33 | NR | Dominant | 2.98(1.14-7.80) |
| Wang  2006 | East Asian | rs2241766 | CHD patients and normal controls | 120/131 | CHD | PCR-RFLP | 0.38 | Yes | Allelic | P<0.05 |
| Jung  2006 | East Asian | rs2241766 | CAD patients and normal controls | 88/68 | CAD | TaqMan genotyping assays  ABI Prism7200 | 0.28 | Yes | Genotypic | P=0.843 |
|  |  | rs1501299 |  |  |  |  | 0.31 | Yes | Genotypic | P=0.954 |
| Lu  2007 | East Asian | rs1501299 | CHD patients and normal controls | 131/135 | CHD | PCR-RFLP | 0.39 | NR | Dominant | 0.37(0.16-0.85) |
| Liang  2008 | East Asian | rs1501299 | CHD patients and normal controls | 100/100 | CHD | PCR-RFLP | 0.50 | NR | Dominant | 2.72(1.08-6.89) |
| Yamada  2008 | East Asian | rs266729 | ACI patients and controls with MetS | 313/971 | ACI | Other: suspension array technology. | 0.23 | Yes | Recessive | 2.14(1.23-3.68) |
| Oguri  2009 | East Asian | rs266729 | MI patients and controls with MetS | 773/1114 | MI | Other | 0.22 | Yes | Dominant | 1.40(1.13-1.74) |
| Chang  2009 | East Asian | rs2241766 | CAD patients and normal controls | 600/718 | CAD | PCR-RFLP | 0.33 | Yes | Additive  TG vs. TT  GG vs. TT | 0.78(0.61-0.98)  0.57(0.37-0.86) |
| Zhang  2009 | East Asian | rs2241766 | CHD patients and normal controls | 205/130 | CHD | PCR-RFLP | 0.30 | Yes |  | Not significant |
|  |  | rs1501299 |  |  |  |  | 0.32 | Yes |  | Not significant |
|  |  | rs266729 |  |  |  |  | 0.25 | Yes | Dominant | 1.62(1.04-2.51) |
| Zhong  2009 | East Asian | rs266729 | CAD patients and normal controls | 198/237 | CAD | TaqMan genotyping assays  ABI Prism377 | 0.22 | Yes | G vs. C  Additive  CG vs. CC  GG vs. CC | 1.24(0.91-1.69)  1.26(0.84-1.89)  1.42(0.67-2.99) |
| Xu  2010 | East Asian | rs2241766 | CHD patients and normal controls | 153/73 | CHD | PCR-RFLP | 0.28 | Yes | Dominant | 2.13(1.03-4.40) |
| Leu  2010 | East Asian | rs1501299 | Stroke patients and normal controls | 80/3330 | Ischemic stroke | Other: matrix-assisted laser desorption ionization time-of-flight | 0.27 | Yes | Additive  TG vs. GG  TT vs. GG | 1.31(0.83-2.07  1.21(0.51-2.87)) |
| Liu  2010 | East Asian | rs2241766 | Stroke patients and normal controls | 302/338 | Ischemic stroke | PCR-RFLP | 0.25 | Yes | Additive  TG vs. TT  GG vs. TT | 1.26(0.87-1.82)  2.16(0.98-4.74) |
|  |  | rs1501299 |  |  |  |  | 0.30 | Yes | Additive  TG vs. GG  TT vs. GG | 1.11(0.76-1.61)  1.07(0.59-1.92) |
|  |  | rs266729 |  |  |  |  | 0.25 | Yes | Additive  CG vs. CC  GG vs. CC | 1.38(0.92-1.94)  2.16(1.12-4.20) |
| Chen  2010 | East Asian | rs2241766 | Stroke patients and normal controls | 357/345 | Ischemic stroke | TaqMan | 0.20 | Yes | Dominant | 1.55(1.14-2.12) |
|  |  | rs266729 |  |  |  |  | 0.25 | Yes | Dominant | 1.13(0.82-1.55) |
| Katakami  2012 | East Asian | rs1501299 | CVD patients and controls with T2D | 213/2424 | CVD | Other | 0.29 | Yes | Allelic | 1.49(1.09-2.05) |
| Al-Daghri  2010 | Saudi | rs2241766 | CAD patients and controls with T2D | 123/295 | CAD | PCR-RFLP | 0.14 | Yes | Dominant | 1.60(1.05-2.5) |
|  |  | rs1501299 |  |  |  |  | 0.39 | Yes | Dominant | 0.96(0.62-1.4) |
| Alireza  2011 | West Asian  Iran | rs2241766 | CHD patients and controls with T2D | 144/127 | CAD | PCR-RFLP | 0.28 | Yes | Recessive | 2.46(1.19-5.09) |
|  |  | rs1501299 |  |  |  |  | 0.32 | Yes | Log-additive model | 0.39(0.22-0.68) |
| Sabouri  2011 | West Asian  Iran | rs2241766 | CAD patients and normal controls | 329/106 | CAD | PCR-RFLP | 0.03 | Yes | Allelic | P<0.001 |
| Boumaiza  2011 | African | rs2241766 | CAD patients and normal controls | 212/104 | CAD | PCR-RFLP | 0.16 | Yes | Genotypic | P=0.762 |
|  |  | rs1501299 |  |  |  |  | 0.37 | Yes | Genotypic | P=0.213 |

ACI= atherothrombotic cerebral infarction; CAD=coronary artery disease; CHD=coronary heart disease; CVD=cardiovascular disease; MI=myocardial infarction; MetS =metabolic syndrome; NR= not report; T2D=type 2 diabetes;
