# Supplementary material for: Association between polymorphisms in the adiponectin gene and cardiovascular disease: a meta-analysis
Source: BMC Med Genet. 2012 May 28;13:40. doi: 10.1186/1471-2350-13-40 (PMC3413575; doi:10.1186/1471-2350-13-40)
Supplement: Additional file 3 — Table S3. Quality assessment for each study included in this meta-analysis. [file 1471-2350-13-40-S3.doc]

**Supplement table 3 -** Quality assessment for each study included in this meta-analysis

| Author | Year | 1 | 2 | 3 | 4 | 5 | 6 | 7 | 8 | 9 | 10 | 11 | 12 | 13 | 14 | 15 | 16 | 17 | 18 | 19 | 20 | 21 | 22 | 23 | 24 | 25 | 26 | 27 | 28 | 29 | 30 | 31 | 32 | 33 | 34 | QS |
| --- | --- | --- | --- | --- | --- | --- | --- | --- | --- | --- | --- | --- | --- | --- | --- | --- | --- | --- | --- | --- | --- | --- | --- | --- | --- | --- | --- | --- | --- | --- | --- | --- | --- | --- | --- | --- |
| Lacquemant | 2003 | 1 | 1 | 1 | 1 |  |  |  |  |  |  | 1 |  |  |  |  |  |  |  |  |  |  |  | 1 |  |  | 1 |  | 1 | 1 |  |  |  |  |  | 9 |
| Lacquemant | 2003 | 1 | 1 | 1 | 1 |  |  |  |  |  |  | 1 |  |  |  |  |  |  |  |  |  |  |  | 1 |  |  | 1 |  | 1 | 1 |  |  |  |  |  | 9 |
| Bacci | 2004 | 1 | 1 | 1 | 1 |  | 1 |  |  |  |  | 1 |  |  |  |  |  |  |  |  |  |  |  |  |  |  | 1 |  | 1 |  |  |  |  |  |  | 8 |
| Ohashi | 2004 | 1 | 1 | 1 | 1 |  | 1 |  |  |  |  | 1 |  |  |  |  |  |  |  |  |  |  |  |  |  |  | 1 |  |  |  |  |  |  |  |  | 7 |
| Stenvinkel | 2004 | 1 | 1 |  | 1 |  | 1 |  |  |  |  | 1 |  |  |  |  |  |  |  |  |  |  |  | 1 |  |  | 1 |  |  |  |  |  |  |  |  | 7 |
| Filippi | 2005 | 1 | 1 | 1 | 1 |  | 1 |  |  |  |  | 1 |  |  |  |  |  |  |  |  |  |  |  | 1 |  |  | 1 |  | 1 |  |  |  |  |  |  | 9 |
| Ru | 2005 | 1 | 1 | 1 | 1 |  |  |  |  |  |  | 1 |  |  |  |  |  |  |  |  |  |  |  |  |  |  | 1 |  |  |  |  |  |  |  |  | 6 |
| Qi | 2005 | 1 | 1 | 1 | 1 |  | 1 |  |  |  |  | 1 |  |  |  |  |  |  |  |  |  |  |  | 1 |  |  | 1 |  | 1 | 1 |  |  |  |  |  | 10 |
| Qi | 2006 | 1 | 1 | 1 | 1 |  | 1 |  |  |  |  | 1 |  |  |  |  |  |  |  |  |  |  |  | 1 |  |  | 1 |  | 1 | 1 |  |  |  |  |  | 10 |
| Gable | 2006 | 1 | 1 | 1 | 1 |  | 1 |  |  | 1 |  | 1 |  | 1 |  |  |  |  |  |  |  |  |  | 1 |  |  | 1 |  | 1 | 1 |  |  |  |  |  | 12 |
| Gable | 2006 | 1 | 1 | 1 | 1 |  | 1 |  |  | 1 |  | 1 |  | 1 |  |  |  |  |  |  |  |  |  | 1 |  |  | 1 |  | 1 | 1 |  |  |  |  |  | 12 |
| Wang | 2006 | 1 | 1 | 1 | 1 |  |  |  |  |  |  | 1 |  |  |  |  |  |  |  |  |  |  |  | 1 |  |  | 1 |  |  |  |  |  |  |  |  | 7 |
| Hegener | 2006 | 1 | 1 | 1 | 1 |  | 1 |  |  | 1 |  | 1 |  | 1 |  |  |  |  |  |  |  |  |  | 1 |  |  | 1 |  | 1 |  |  |  |  |  |  | 11 |
| Hegener | 2006 | 1 | 1 | 1 | 1 |  | 1 |  |  | 1 |  | 1 |  | 1 |  |  |  |  |  |  |  |  |  | 1 |  |  | 1 |  | 1 |  |  |  |  |  |  | 11 |
| Jung | 2006 | 1 | 1 | 1 | 1 |  | 1 |  |  |  |  | 1 |  |  |  |  |  |  |  |  |  |  |  | 1 |  |  | 1 |  |  |  |  |  |  |  |  | 8 |
| Pischon | 2007 | 1 | 1 | 1 | 1 |  | 1 |  |  |  |  | 1 |  | 1 |  |  |  |  |  |  |  |  |  | 1 |  |  | 1 |  | 1 | 1 |  |  |  |  |  | 11 |
| Lu | 2007 | 1 | 1 | 1 | 1 |  | 1 |  |  |  |  | 1 |  |  |  |  |  |  |  |  |  |  |  |  |  |  | 1 |  |  |  |  |  |  |  |  | 7 |
| Liang | 2008 | 1 | 1 | 1 | 1 |  |  |  |  |  |  | 1 |  |  |  |  |  |  |  |  |  |  |  |  |  |  | 1 |  |  |  |  |  |  |  |  | 6 |
| Yamada | 2008 | 1 | 1 | 1 | 1 |  | 1 |  |  |  |  | 1 |  |  |  |  |  |  |  |  |  |  |  | 1 |  |  | 1 |  |  | 1 |  |  |  |  |  | 9 |
| Oguri | 2009 | 1 | 1 | 1 | 1 |  | 1 |  |  | 1 |  | 1 |  |  |  |  |  |  |  |  |  |  |  | 1 |  |  | 1 |  |  | 1 |  |  |  |  |  | 10 |
| Chang | 2009 | 1 | 1 | 1 | 1 |  | 1 |  |  |  |  | 1 |  |  |  |  |  |  |  |  |  |  |  | 1 |  |  | 1 |  |  | 1 |  |  |  |  |  | 9 |
| Zhang | 2009 | 1 | 1 | 1 | 1 |  | 1 |  |  |  |  | 1 |  |  |  |  |  |  |  |  |  |  |  | 1 |  |  | 1 |  |  |  |  |  |  |  |  | 8 |
| Zhong | 2009 | 1 | 1 | 1 | 1 |  | 1 |  |  |  |  | 1 |  | 1 |  |  |  | 1 |  |  |  |  |  | 1 |  |  | 1 |  |  |  |  |  |  |  |  | 10 |
| Foucan | 2009 | 1 | 1 |  | 1 |  | 1 |  |  |  |  | 1 |  |  |  |  |  |  |  |  |  |  |  | 1 |  |  | 1 |  |  |  |  |  |  |  |  | 7 |
| Caterina | 2010 | 1 | 1 | 1 | 1 |  | 1 |  |  | 1 |  | 1 |  | 1 |  |  |  | 1 |  |  |  |  |  | 1 |  |  | 1 |  | 1 | 1 |  |  |  |  |  | 13 |
| Xu | 2010 | 1 | 1 | 1 | 1 |  | 1 |  |  |  |  | 1 |  |  |  |  |  |  |  |  |  |  |  | 1 |  |  | 1 |  |  |  |  |  |  |  |  | 8 |
| Al-Daghri | 2010 | 1 | 1 | 1 | 1 |  | 1 |  |  |  |  | 1 |  |  |  |  |  |  |  |  |  |  |  | 1 |  |  | 1 |  |  |  |  |  |  |  |  | 8 |
| Prior | 2010 | 1 | 1 |  | 1 |  | 1 |  |  |  |  | 1 |  |  |  |  |  |  |  |  |  |  |  | 1 |  |  | 1 |  |  |  |  |  |  |  |  | 7 |
| Chiodini | 2010 | 1 | 1 | 1 | 1 |  | 1 |  |  |  |  | 1 |  | 1 |  |  |  |  |  |  |  |  |  | 1 |  |  | 1 |  |  | 1 |  |  |  |  |  | 10 |
| Rodriguez | 2010 | 1 | 1 | 1 | 1 |  |  |  |  |  |  | 1 |  |  |  |  |  | 1 |  |  |  |  |  | 1 |  |  | 1 |  | 1 |  |  |  |  |  |  | 9 |
| Leu | 2010 | 1 | 1 | 1 | 1 |  | 1 |  |  | 1 |  | 1 |  |  |  |  |  |  |  |  |  |  |  | 1 |  |  | 1 |  |  | 1 |  |  |  |  |  | 10 |
| Liu | 2010 | 1 | 1 | 1 | 1 |  | 1 |  |  |  |  | 1 |  |  |  |  |  |  |  |  |  |  |  | 1 |  |  | 1 |  | 1 |  |  |  |  |  |  | 9 |
| Chen | 2010 | 1 | 1 | 1 | 1 |  | 1 |  |  |  |  | 1 |  |  |  |  |  |  |  |  |  |  |  | 1 |  |  | 1 |  |  |  |  |  |  |  |  | 8 |
| Sabouri | 2011 | 1 | 1 | 1 | 1 |  | 1 |  |  |  |  | 1 |  |  |  |  |  |  |  |  |  |  |  | 1 |  |  | 1 |  |  |  |  |  |  |  |  | 8 |
| Alireza | 2011 | 1 | 1 | 1 | 1 |  | 1 |  |  |  |  | 1 |  |  |  |  |  |  |  |  |  |  |  | 1 |  |  | 1 |  | 1 | 1 |  |  |  |  |  | 10 |
| Boumaiza | 2011 | 1 | 1 | 1 | 1 |  | 1 |  |  |  |  | 1 |  | 1 |  |  |  |  |  |  |  |  |  | 1 |  |  | 1 |  | 1 |  |  |  |  |  |  | 10 |
| Katakami | 2012 | 1 | 1 | 1 | 1 |  | 1 |  |  | 1 |  | 1 |  |  |  |  |  | 1 |  |  |  |  |  | 1 |  |  | 1 |  |  | 1 |  |  |  |  |  | 11 |

Checklist of conditions for quality assessment

| **Study information** | |
| --- | --- |
| 1 | A detailed description of the study design and its implementation |
| 2 | The source of cases and controls or cohort members, if based on cohort design |
| 3 | Methods for ascertaining and validating affected or unaffected status and reproducibility of classification |
| 4 | Participation rates for cases, controls or cohort members |
| 5 | Presentation of case and control selection in a flow chart |
| 6 | Initial table comparing relevant characteristics of cases and controls |
| 7 | Success rate for DNA acquisition |
| **Data issue** | |
| 8 | Statement on availability of results and data |
| 9 | Links to supplemental online resources and database accession numbers |
| **Genotyping and quality control procedures** | |
| 10 | Sample tracking methods, such as barcoding, to ensure accuracy of analysis |
| 11 | Description of genotyping assays and protocols |
| 12 | Description of genotyping calling algorithm |
| 13 | Genotype quality control design for samples |
| 14 | External control samples from standard accepted sets (such as HapMap) |
| 15 | Internal control samples |
| 16 | Assay and DNA quality metrics by locus, sample, plate or ‘batch’ |
| 17 | Assay call rates |
| 18 | Average error rates estimated by internal duplicates or external samples |
| 19 | Assay reproducibility: concordance for performance of extraction, aliquoting and assay reproducibility |
| 20 | Concordance with published or previously generated genotypes |
| 21 | Mendelian consistency checks if related individuals are present |
| 22 | Detection of inconsistent or cryptic relatedness in study subjects |
| 23 | Evaluation of deviations from Hardy–Weinberg proportions separately in cases and controls |
| 24 | Assessment of population heterogeneity, including |
|  | 24(1)Average or median value of chi-square and full distribution (0.5 score) |
|  | 24(2)Q–Q plots of chi-square analysis and *P*values (0.5 score) |
| 25 | Validation of most critical results on an independent genotyping platform |
| **Results** | |
| 26 | Analysis methods in sufficient detail to reconstruct the analytical approach |
| 27 | Description of any pre-analysis weighting scheme for selecting variants for replication |
| 28 | Simple single-locus and multi-marker (haplotype) association analyses |
| 29 | Genetic models tested |
| 30 | Graphical display of genotype clustering for assays of high interest |
| 31 | Verification of results at highly correlated loci |
| 32 | Discussion of choice of threshold for significance |
| 33 | Significance of any known ‘positive controls’ |
| 34 | Consistency of results before and after application of quality control filters |
